# Experiences and Needs of Leaders Supporting Multilingual ABA Staff: A Qualitative Exploration

**DOI:** 10.1007/s10803-025-06816-w

**Published:** 2025-04-10

**Authors:** Melanie R Martin Loya, Hedda Meadan, Elaine M. Gilmartin

**Affiliations:** 1 University of California Davis, MIND Institute, Davis, USA; 2 University of North Carolina at Charlotte, Charlotte, USA; 3 University of Illinois Urbana Champaign, Champaign, USA

**Keywords:** Autism, Multilingualism, Bilingualism, Supervision, Behavior analysis, Service delivery

## Abstract

Heritage-language-speaking caregivers of autistic children in the United States (U.S.) have encountered barriers when attempting to access multilingual support for their children. Multilingual autism providers, such as professionals in Applied Behavior Analysis (ABA), have also reported challenges in providing multilingual care, such as a lack of training opportunities. Little is known about the experiences and needs of leaders in ABA who support multilingual direct care staff. Therefore, the current study aimed to answer the research question: What are the experiences and needs of ABA leaders in autism care related to supporting multilingual ABA staff who work with multilingual autistic children and their families? This exploratory qualitative study used semi-structured focus groups and interviews with monolingual and multilingual behavior analysts who had experience supporting multilingual ABA staff. Transcripts were analyzed using reflexive thematic analysis. The 14 participants were a diverse sample of U.S.-based monolingual, multilingual, autistic, and non-autistic leaders in ABA with experience supervising multilingual ABA staff. Two major themes were generated from the data set: (1) Diverse Experiences: Organizational Policies and Actions Impacting Multilingual Staff, and (2) Building Community and Creating Solutions. Participants shared experiences and challenges supervising multilingual ABA staff. Implications highlight the need to closely examine organizational policies to prevent discrimination and spotlight the need for more research to be conducted in this area.

**Supplementary Information:**

The online version contains supplementary material available at 10.1007/s10803-025-06816-w.

Multilingualism is common around the world and has been described as impactful as shaping “cells, selves, and societies” (Chung-Fat-Yim et al., 2023, p. 321; Marian, 2023). Autistic children from multilingual or heritage-language-speaking homes (hereafter referred to as multilingual autistic children) are a growing population that commonly receives care from disability support providers, such as Applied Behavior Analysis (ABA) professionals, special educators, and speech-language pathologists. Multilingual autistic children are defined as those with families who communicate using a heritage language (i.e., any non-dominant language in a given society; Kelleher, 2010) or communicate in two or more languages^1^. This definition also includes children who may be emerging communicators and/or use alternative or augmentative forms of communication. While broad, this definition encapsulates a population of children and families that are underrepresented in research (Steinbrenner et al., 2022) and often encounter barriers to service access in education (Barrera-Lansford & Sánchez, 2024) and allied health fields such as ABA (Baires et al., 2023; Lim et al., 2021) and speech-language pathology (The American Speech-Language-Hearing Association [ASHA], 2023). These disparities have led to decreases in the utilization of specialized services (Cohen et al., 2023; Zuckerman et al., 2017); therefore, many children and their families do not receive the services recommended to support their development and growth. Indeed, multilingual autistic children and their families have been shown to benefit from receiving care tailored to their cultural and linguistic needs (Gilhuber et al., 2023; Trelles & Castro, 2019) and they have reported negative consequences to receiving English-only recommendations from professionals (Reimann & Ratto, 2023).

First, it is important to emphasize that extant research does not support the idea that multilingual autistic children are better served in monolingual (English-only) environments (Beauchamp et al., 2023; Gilhuber et al., 2023). In addition, heritage-language-speaking families in the U.S. have been found to have greater difficulty accessing support services (Zuckerman et al., 2017), and some child-level negative impacts have also been reported. For example, bilingual behavior analysts reported witnessing increases in behaviors such as aggression and self-harm when autistic children from heritage-language-speaking families received English-only care (Martin Loya & Meadan, 2024b), and some studies have demonstrated increases in social behaviors when care is provided in autistic children’s heritage languages compared to English (Lang et al., 2011; Lim & Charlop, 2018). In non-autistic populations, researchers have also found that children’s proficiency in their parents’ heritage languages predicted stronger bonds between parent and child (deSouza et al., 2023; Müller et al., 2020; Oh & Fuligni, 2010), and heritage-language maintenance across generations may contribute to stronger social cohesion, which has been found to be a protective factor that improves mental health outcomes of immigrant populations in the U.S. (Elshahat & Moffat, 2022; Escobar et al., 2000; Leong et al., 2013; Garcini et al., 2021). These findings affirm efforts that support heritage language maintenance within families, which includes linguistically tailoring autism care.

Multilingual autistic children may also benefit from care tailored to their linguistic profiles with improved executive functioning (Ratto et al., 2020, 2022; Sharaan et al., 2022), social and language skills (Beauchamp et al., 2020; Ge et al., 2024; Siyambalapitiya et al., 2022; Zhou et al., 2019), and play skills (Lim & Charlop, 2018). In addition, caregivers of multilingual autistic children have reported a desire to maintain their heritage languages with their children (Papoudi et al., 2021) but encounter challenges such as receiving inaccurate information from professionals (e.g., the myth that multilingualism can be harmful to autistic children; Reimann & Ratto, 2023). Finally, we know that autistic individuals have reported valuing and benefiting from multilingualism (Digard et al., 2020, 2022; Nolte et al., 2021), and that multilingualism has been highlighted as an important area needing additional attention in autism research (Davis et al., 2022).

While multilingualism has been identified as important for the autism community, multilingual providers are in high demand but in short supply. We were unable to locate national data related to the availability of multilingual special educators, but we know that special educators and multilingual educators as distinct groups have experienced decades-long shortages in the United States ([U.S.]; National Center for Education Statistics, 2023). In addition, special educators have reported that supporting linguistically diverse children is an area where they need more training and support (Fowler et al., 2019). In allied professions, shortages have been reported for multilingual ABA providers (Martin Loya & Meadan, 2024a), speech-language pathologists (ASHA, 2023), school psychologists (Olvera & Olvera, 2015), and clinical psychologists (Diaz-LePage et al., 2024). Summarily, multilingualism in disability care and research is an area with room for exploration and much improvement, including within the field of ABA.

## ABA and Bilingualism

ABA is the science of behavior change, and Board Certified Behavior Analysts (BCBAs) are graduate-level providers with expertise to design, conduct, and supervise others who are providing care based on the principles of ABA. Most BCBAs work in a tiered-service-delivery model, where BCBAs supervise direct care providers (e.g., Board Certified Assistant Behavior Analysts or Registered Behavior Technicians [RBTs]) who have received training to implement behavioral treatment plans (Behavioral Health Center of Excellence, 2017). Little empirical work has been published related to multilingualism within ABA practice. However, we know that most BCBAs identify as White, female, and live in the U.S., which leads us to assume that the average BCBA is a monolingual English speaker (Behavior Analyst Certification Board [BACB], n.d.). We also know that multilingual caregivers of autistic children have reported positive outcomes of their children receiving ABA services (Castro-Hostetler et al., 2023; Cohen et al., 2023; Rosales et al., 2021), but also difficulties related to service access, funding, and a lack of multilingual support (Cohen et al., 2023). These experiences are echoed by caregivers of autistic children in autism-support services broadly (e.g., in early intervention and special education services; Durán et al., 2022; Papoudi et al., 2021). Notably, caregivers’ experiences and needs are directly linked to the experiences and skills of the providers (Gordillo et al., 2022).

The multilingual ABA workforce has been explored much less in research and has primarily focused on Latino/Hispanic^2^ populations. In practice, multilingual BCBAs have reported difficulties in the workplace, such as a need for more translated resources and a desire for greater understanding and recognition from colleagues and families (Martin Loya & Meadan, 2024a). However, they have also reported experiencing many positive aspects of providing heritage-language care to multilingual autistic children, such as stronger connections with families and great joy and value in providing a highly sought-after service (Martin Loya and Meadan, 2024b). We also know there has been an increase in Latina women publishing behavior analytical research (Li et al., 2024), still, their numbers do not reflect the number of Latinas within the field of ABA nor in the U.S. in general (BACB, n.d.; Vespa et al., 2020). In addition, Latino/Hispanic students enrolled in ABA programs have reported barriers to entering the field (Fernandez et al., 2024) and are not necessarily reflective of Latinos/Hispanics throughout the U.S. in terms of levels of education, religiosity, or other cultural norms (Jimenez-Gomez et al., 2024). Indeed, the multilingual ABA workforce, including Latinos/Hispanics, is diverse, with distinct backgrounds, experiences, joys, and challenges.

To date, no research has explored what it is like to support multilingual ABA staff – from the perspective of either monolingual or multilingual BCBA leaders. This is an important step toward building our understanding of the ABA workforce to improve it and create effective and equitable pathways toward employment for future autism professionals who have the language skills to support the growing population of multilingual autistic children in the U.S. Therefore, the purpose of the present study was to examine the perspectives of ABA leaders with experience supervising or mentoring multilingual staff who provide direct care to multilingual autistic children and their families to better understand workforce experiences and needs.

## Method

This exploratory qualitative study used focus groups and interviews to answer the research question: What are the experiences and needs of ABA leaders in autism care related to supporting multilingual ABA staff who work with multilingual autistic children and their families? This study was part of a larger exploratory sequential mixed methods experimental design (Creswell, 2022; Greene, 2007). This article showcases phase one, the qualitative component, that was used to develop a professional development for BCBAs (see Martin Loya et al., 2025).

### Theoretical Framework and Researchers’ Identities

This work was strongly influenced by Critical Race Theory (CRT) and Disability Critical Race Theory ([DisCrit]; Annamma et al., 2018; Connor et al., 2016; Crenshaw, 1989) and was conducted under a social constructionism epistemological stance (Burr, 2015). CRT and DisCrit encourage us to examine the intersections of our multiple historically marginalized identities rather than viewing them in isolation. For example, researchers have found that Latino/Hispanic BCBAs’ generation in the U.S. impacts their experiences and highlights the caution one must take not to generalize groups of people (e.g., Spanish-speaking BCBAs who are first generation in the U.S. reported more experiences of discrimination, and Spanish-speaking BCBAs who are second and third generation reported needing more translation and interpretation support; Martin Loya & Meadan, 2024a). This work is informed by CRT and DisCrit as evidenced throughout the study, including the purposeful recruitment and inclusion of a range of BCBAs who work in distinct settings, speak multiple languages, and have various identities related to autism, including autistic BCBAs and family members.

The research team included three BCBAs who are all multilingual (English, Hebrew, and Spanish speakers), with various professional experiences (e.g., primary and higher education and clinical ABA), and experienced in qualitative research methods (i.e., all authors had conducted and published qualitative research prior to the present study). This study was primarily conceptualized, conducted, and written by the first author, who also has an autistic brother with multiple disabilities. Lastly, the first and third authors also consider themselves insider-outsiders to the participant population (i.e., we both practiced as bilingual BCBAs in a tiered service delivery model for years prior to entering academia and could relate to many of the stories shared by our participants; Dwyer & Buckle, 2009); we recognize this can create helpful relationships in qualitative research but can also pose challenges which necessitated careful reflexivity and self-awareness in the analysis process (Ademolu, 2024). In alignment with social constructionism, we believe meaning is socially produced and our various identities and biases were ultimately an asset in the present study (Braun & Clarke, 2006, 2022). Steps taken to enhance credibility and trustworthiness are further explored below.

### Recruitment and Participants

Recruitment was purposeful and targeted via (1) social media advertisements in public social media spaces that targeted behavior analysts and (2) chain-referral sampling (i.e., snowball sampling). Participant inclusion criteria required (a) BCBAs in good standing per the BACB, (b) having completed a BACB-approved 8-hour supervisor training, (c) being at least one-year post-certification, (d) primarily serving autistic children and their families, (e) having experience providing mentorship or supervision to multilingual ABA staff who practice multilingually or in their non-English language, currently or in the past, and (f) live and work in the United States.

Thirty people filled out the Google form screener, 28 of whom were eligible to participate, and 14 of those eligible individuals completed interviews or participated in focus groups. Anecdotally, some people who filled out the screener but did not participate reported scheduling difficulties as their reason for not participating and others did not respond to any contact attempts. Recruitment concluded when saturation was achieved (i.e., saturation for the purpose of developing the intervention for phase two of the larger mixed-methods study; Tracy, 2019). All 14 participants identified as female and had at least five years of experience working in ABA. Participants included those multilingual in English and Spanish, American Sign Language, Persian, and monolingual English-speaking BCBAs. Participants were mostly Latino/Hispanic (*n* = 7), followed by White or European American (*n* = 6), Black or African American (*n* = 2), Indigenous (*n* = 1), and Middle Eastern or North African (*n* = 1); some participants identified with more than one racial or ethnic group. A few participants had no additional connections to autism outside of their work (*n* = 5), but participants also reported being close family members of autistic individuals (e.g., parents, siblings, or aunts; *n* = 4) and close friends of autistic individuals (*n* = 4). In addition, two participants identified as autistic, and one participant chose not to share. Additional demographic information can be found in Table 1.

**Table 1:** Participant demographics

	Participants (*N* = 14)
**Age in Years –** ***M*** **(range)**	*35* (30–45)
**Years of Experience in ABA –** ***M*** **(range)** ^**a**^	*13.5* (5–25)
**Race and/or Ethnicity** ^**b**^	
Latino/Hispanic	7
White or European American	6
Black or African American	2
Indigenous	1
Middle Eastern or North African	1
**U.S. State of Primary Practice**	
California	3
Texas	3
Arizona	2
Connecticut	1
Florida	1
Hawaii	1
Illinois	1
Mississippi	1
Virginia	1
**Highest Degree Earned –** ***M*** **(%)**	
Master’s Degree	*8* (57)
Doctoral Degree	*6* (43)
**Role at Work**	
Case Management in Tiered Service Delivery Model	6
Higher Ed. or Professional Supervision	3
Clinical Director - Local	2
Clinical Director - Regional	2
Business Owner	1
**Languages with Proficiency** ^**c**^	
Bilingual - Spanish	7
Monolingual - English	5
Bilingual - American Sign Language	1
Bilingual - Persian	1
**Connections to Autism** ^**d**^	
Work only	5
Close Family Member	4
Close Friend	4
Autistic	2
Preferred not to share	1

Note. ^a^Years of experience in ABA included time spent as a behavior technician, if applicable; ^b^Numbers do not equal to 14 because some participants identified as having more than one race and/or ethnicity; ^c^All multilingual participants were also fluent in English. ^d^Numbers do not equal to 14 because some participants held multiple identities and relationships related to autism

### Procedures

Interested participants accessed the Google screening form by following the URL on the recruitment flier, email, or post. After completing the screening form, they automatically advanced to the consent form and then a short demographic questionnaire. The scheduling and assignment of the focus groups were based on participant availability, and individual interviews were offered to those with scheduling conflicts who could not attend focus groups. Two one-to-one interviews and four focus groups were conducted over Zoom. All focus groups included the third author, who acted as a note-taker and accommodation support by inputting questions into the chat feature so participants could hear and read the questions. Individual interviews did not include a note-taker. Individual interviews were 26 min and 19 min. Focus group durations ranged from 61 to 74 min and averaged 66 min, with participants ranging from two to four participants per focus group. Additional accommodations were offered to encourage participation and accessibility for neurodiverse populations, including access to (a) respond to focus group questions individually via email or in an individual interview, (b) review the focus group interview template in advance of the focus group, and (c) respond to focus group questions using the chat feature in Zoom. Accommodation plans were created by consulting the literature (Kim et al., 2022; Nicolaidis et al., 2019) and through personal correspondence with autistic colleagues. The two autistic participants declined any additional accommodations besides the standard caption feature in Zoom.

Focus group and interview audio were transcribed using the transcription feature in Microsoft Word and reviewed manually to ensure accuracy. All transcripts were verified within one week of the focus group or interview, and member check (i.e., short summary) emails were sent to all participants (Brantlinger et al., 2005). Member checks were used to verify the accuracy or inaccuracy of the authors’ initial impressions of the participants’ contributions. Participants provided minor corrections to the member check summaries. The verified transcripts were collaboratively analyzed by the authors. All focus groups were conducted in August 2023, and the individual interviews were conducted in September 2023. As a token of appreciation, all participants were provided a $50 e-gift card upon completion of all study components.

### Data Collection

Data sources included an individual demographic questionnaire and semi-structured individual and focus group interviews.

#### Instruments

The screener and demographic questionnaire (see the Supplementary Information [SI]) included questions related to racial and ethnic identity, language(s) spoken, work and certification history, and personal connections to autism outside participants’ professions. The question related to their connection(s) to autism was included in light of recent literature highlighting the autistic community’s desire to know more about who is involved with research (Nicolaidis et al., 2019). This question was crafted and reviewed in consultation with one autistic researcher and four BCBAs who were not autistic.

The semi-structured focus group protocol (SI) focused on understanding participants’ experiences and needs related to supporting multilingual ABA staff and was informed by Martin Loya & Meadan’s findings (2024a; 2024b). Focus groups were chosen as the data collection method to encourage participant interaction. The focus group protocol was reviewed by three BCBAs, which resulted in minor edits for clarity. In addition, a fidelity checklist was used in all focus groups and interviews to ensure consistency across the groups and can be found in the SI. The fidelity checklist was completed by the first author and the note-taker for all focus groups. The same protocol was used for the two individual interviews, but irrelevant instructions were omitted (e.g., guidelines for maintaining peers’ privacy in the focus groups). All substantive questions and information were the same between focus group and individual interviews.

### Data Analysis

The research team utilized reflexive thematic analysis (Braun & Clarke, 2006, 2022; Creswell & Creswell, 2018) to generate themes. First, we independently familiarized ourselves with the data while cleaning transcripts and creating member check summaries. Then, we read through all focus group transcripts independently to build ideas and take note of general topics and responses covered in focus groups and initial impressions (Saldaña, 2021) and discussed our general thoughts and impressions of the data set. The team then independently re-read transcripts and independently coded them sentence by sentence or passage by passage, depending on the content of the transcripts. Codes are defined as short phrases or single words to describe the content of the sentence or passage. Next, we met to compare agreements and disagreements in our initial coding. This process was repeated until all transcripts were reviewed and coded once. Then, the first author created an initial codebook that included definitions, exemplary quotes, and categorized codes under potential themes. Preliminary themes were generated by the first author after creating a first draft of the codebook. Next, the full research team reviewed the codebook using a consensus and iterative approach until all themes across the focus groups and individual interviews were adequately captured within the codebook (Braun & Clarke, 2006, 2022; Saldaña, 2021; an abbreviated codebook is available in the SI). All transcripts were then re-coded using the final version of the codebook in Dedoose software (2022) to capture themes across the different focus groups and interviews. Various quality indicators were addressed in this research and are outlined in the SI.

### Findings

The 14 multilingual and monolingual behavior analysts who participated in the focus groups and interviews shared their experiences and needs related to supporting multilingual ABA staff. The findings presented include all participants’ experiences supporting multilingual staff as well as the multilingual participants’ own experiences of being members of a larger team. Overall, participants highly value multilingualism, believe providing services in other languages is important, and they share many challenges and needs related to working multilingually or supporting multilingual staff and families. Through reflexive thematic analysis, the current study illuminated novel findings that aligned with two major themes and two sub-themes that were generated from the dataset. The first major theme is: Diverse Experiences: Organizational Policies and Actions Impacting Multilingual Staff, with two sub-themes, (1) Steps in the Right Direction, and (2) Barriers to Progress. The second major theme is: Building Community and Creating Solutions. These themes and sub-themes are included in Table 2 and explored in detail below. Pseudonyms are used to protect participants’ identities. **[**Table 2**]**

**Table 2:** Themes and subthemes

Title	Description
Diverse Experiences: Organizational Policies and Actions Impacting Multilingual Staff	This major theme includes participants’ stories related to concrete examples of how organizational policies (or a lack thereof) contributed to their ability (or inability) to provide high-quality multilingual care or support a multilingual staff member.
• Steps in the Right Direction	This sub-theme includes specific examples of positive actions or policies supported by participants’ employers.
• Barriers to Progress	This sub-theme includes specific examples of harmful actions or policies supported by participants’ employers.
Building Community and Creating Solutions	This major theme includes participants’ stories related to actions they have taken that went beyond their typical job duties.

### Diverse Experiences: Organizational Policies and Actions Impacting Multilingual Staff

Participants shared stories that demonstrated the impacts of workplace culture and policy on their actions and experiences in supporting multilingual staff in the workplace, both positive and negative.

### Steps in the Right Direction

Participants described a range of organizational policies and actions, further described below, that they believed had positive impacts on their ability to work multilingually and support multilingual staff, including (a) providing support for translation and interpretation, (b) purposeful hiring, (c) culturally and linguistically matching staff members to families, and (d) organization-wide acceptance and celebration of culture and language.

Participants identified that receiving translation (i.e., written language support, such as translated assessments) or interpretation (i.e., in-vivo language support, such as a professional attending and interpreting during Individualized Education Program meetings) support in their places of work was highly beneficial to supporting multilingual staff and providing multilingual services. However, participants noted that translation and interpretation should be viewed as the bare minimum, and they also identified many challenges with existing translation and interpretation services, such as scheduling difficulties (e.g., cancelations or ‘no shows’) or a lack of specialized knowledge in ABA and autism. Participants described these services as better than nothing, but insufficient. For example, one multilingual participant, Jackie, describing the quality and consistency of the translation and interpretation services saying, “They try their best. I don’t wanna say it’s consistent, but… They try their best with it.”

Another action described by participants as helpful is when companies engage in purposeful hiring to increase the number of multilingual individuals on staff. For example, Karee, who is also multilingual, described how her place of work hired an administrative position that functioned as a community liaison to connect families with additional resources within and outside of their organization. She said:

[The company] hired someone to facilitate community resources and they made sure to hire this person that had, I think she speaks 3 languages… And so that’s something I saw implemented that helps a lot. So now they’re referring families to speak to that individual to seek out resources in the community. I felt that was a good step in the right direction.

Sophie, a monolingual BCBA, shared a similar experience where her company hired a multilingual RBT with built-in “flex time” so they could attend meetings and provide language support to colleagues and families, as needed.

When companies purposefully hire multilingual staff, they can also engage in what participants described as “culture matching,” where the company purposefully attempts to match staff and families by culture and language whenever possible^3^. Several participants described culture matching as a positive practice to support multilingual staff and families. Vicki, another monolingual participant, compared the difference between working through an interpreter and working with a multilingual RBT, stating:

I think that it is incredibly helpful to have an RBT, or a person working with [the family] directly, who is from the same culture as the family… I’ve worked in both. I’ve worked [with an interpreter] in a setting where the family spoke a language that was very unique. There weren’t that many people who spoke it in the area… And I found that it was much more difficult to get that connection… but then I’ve also worked with an RBT who spoke the language that the family did, and there’s much more buy-in from the family, much more connection, and I think that’s very important and useful.

However, one multilingual participant, Addy, noted that while culture or language matching can be “helpful,” it’s not always sufficient. Addy continued, “Just because I speak Spanish, or I might have a similar background as someone doesn’t mean I’m an expert translator or that I’m able to communicate our profession effectively in my native language or in the family’s native language.”

Generally, participants also stressed that an acceptance and celebration of cultures and languages was important for creating a positive work environment that supports multilingual staff members and families. Barbra, who is multilingual, described how her workplace was very supportive of providing care in other languages and supporting multilingual staff. She noted that they serve a largely Latino/Hispanic population, and the clinical director and multiple staff members speak both English and Spanish and are Latino/Hispanic. She said, “I do work for a smaller agency that is local, so it’s not a big company that I’m fighting against,” which contrasts with the experiences of many participants who described working for larger national companies, some of which have English-only policies. Overall, participants felt that companies needed to do much more to achieve an equitable workplace for multilingual staff and provide the highest quality care for heritage-language-speaking families, and only two participants described workplaces as very positive or supportive of multilingual staff. Notably, many of the positive practices shared by participants were generally viewed as insufficient, and participants cautioned that some of these practices could have unintended negative consequences.

### Barriers to Progress

Participants described situations as hindering their ability to provide the highest quality multilingual care and supervision of multilingual staff, including (a) a shortage of multilingual personnel, (b) lack of specific training on multilingual autism care, (c) the tokenization and misunderstandings monolingual colleagues often have of their multilingual peers, and (d) the lack of appropriate monetary compensation for multilingual staff. First, a shortage of multilingual staff was consistently mentioned by both monolingual and multilingual participants as a serious barrier to being able to provide the best care. For example, after sharing the story of hiring the multilingual RBT with flex time to provide language support, Sophie continued, stating:

Unfortunately… we have a certain number of hours [available to the multilingual RBT] and so we can’t take any more diverse clients. We don’t take clients in if we do not have reliable communication with the family. And that is terrible. It is discriminatory. If we could provide quality clinical services to these clients, we would.

Sophie and other participants lamented their inability to provide enough heritage language services for families in need.

In addition to a shortage of multilingual personnel, participants noted how a lack of specific training on how to provide services in other languages was a barrier to progress. Notably, none of the participants reported receiving training specific to providing multilingual care within ABA practice. Those who had received some training related to multilingualism had received it outside of the scope of ABA (e.g., in their training to become clinical psychologists or in undergraduate training in other human services fields). Participants often described receiving training that focused on cultural competence or cultural humility but noted that these trainings were never related to supporting staff members, only children and families. As a result of this lack of training, many multilingual participants described scenarios of “winging it” and being thrust into responsibilities they were unprepared to handle. For example, Addy recalled her time working as an RBT:

I was serving as [an interpreter], and I’m sure I said a lot of things incorrectly, you know, because I was kind of just like, not a professional [interpreter], first of all, but also, not a BCBA that could really effectively communicate some of the things that [my BCBA] was trying to communicate to the family. And so, I think that adds to the cycle of disparity that we have with marginalized families, immigrant families, who can’t really contact the direct service, the direct person who’s managing the program in their home.

Like Addy, most multilingual participants described being the only multilingual staff member in their current and former places of work or being one of only a few.

The experience of being the only multilingual person or one of a few resulted in what both monolingual and multilingual participants described as the “tokenization” of multilingual staff. One exchange in a focus group between a monolingual BCBA and a multilingual BCBA exemplified this scenario. Helen, a monolingual BCBA, discussed working in a clinical setting where the one Spanish speaker’s work was often interrupted by colleagues:

It seemed like people would just catch [the multilingual staff member] in the hall and sort of interrupt her other activities like, “Hey, can I borrow you for a second [to help interpret]?…,” And she eventually said, “I would appreciate it being more of a scheduled thing… and not just being always available to do that, as a ‘service’ to the company.” Essentially, because there was no context in which English speakers, at any given moment, somebody would come to your office and be like, “Hey, I need you right now.” Interrupting your activities to help them with something else.

In response to Helen, Nereida, who is multilingual, described how she also felt tokenized and pigeon-holed by her monolingual colleagues, and she recommended that leadership in organizations must be made aware of:

The issue of certain staff being tokenized or only being consulted for [multilingual] situations, is really important because… We may have good intentions, right? Of just only going to that [multilingual] colleague [for language help], but the impact that it has for them should take precedence over our intention… So, being aware of that, and reinforcing when that person sets boundaries, right? That’s super important. And then also communicating [those expectations] to [other] staff.

Nereida and other participants recognized the power that organizational climate and expectations can have on staff members’ behaviors and the impacts of their behavior on colleagues.

Finally, most multilingual participants shared that they were not and had never been financially compensated for providing multilingual services. One monolingual participant, Jenny, shared that her multilingual colleagues in her place of work were financially compensated, though this seems to be an exception. When asked to choose a single action that could improve multilingual care and support of multilingual staff, many participants stated that appropriately compensating multilingual staff would be the most powerful action for organization leadership to take. Overall, for this theme, participants reported that a shortage of multilingual personnel, the lack of specific training on multilingual autism care, the tokenization and misunderstandings monolingual colleagues have of their multilingual peers, and the lack of appropriate financial compensation for multilingual staff were all barriers to advancing high-quality multilingual care for autistic children receiving ABA services.

### Building Community and Creating Solutions

This standalone theme includes stories of individuals’ actions taken to support multilingual practice and staff that went “above and beyond” the expectations or confines of their workplace. Stories shared that we categorized under this theme involved (a) creating new positive spaces, (b) creativity and the cultivation of strong communication with multilingual colleagues and families, and (c) purposeful supervision practices toward multilingual staff. The stories captured within this theme are conceptualized as the positive side of the experiences of ‘winging it,’ as discussed in the sub-theme, Barriers to Progress (e.g., recall Addy describing being forced into an interpreter role despite being unprepared and unqualified to do so). Under the current theme, the necessity of flexibility in multilingual practice is transformed into a vehicle of innovation and positive actions. For example, one multilingual participant, Danica, describes her career-long journey in advocacy and when, as a younger multilingual clinician starting in the field, she could not find a workplace that suited her desire to advance inclusion and accessibility for multiple marginalized families:

[As a younger clinician, ] I knew intuitively that there were issues [in linguistic equity in ABA] and I was there saying like, “Why isn’t anyone else talking about this?” And I was punished a lot for talking. For saying the thing that no one else was saying and it impacted the way that I showed up as a clinician. It didn’t stop me from doing that. But I didn’t make enough movement in advocacy… And so, I stopped doing that for that company. And then I’d go find another company, and then I’d do it there, until eventually I developed my own company where I have the freedom to support families the way that I felt like they needed to be supported, because I was working primarily in Black and brown communities.

Danica’s experiences as a multilingual staff member at unsupportive companies inspired her to create her own company, where inclusion and accessibility for multiple marginalized families were built into the company ethos. Other multilingual participants also described scenarios where they created something new to support each other, on a smaller scale. For example, Amanda, who is multilingual, described having a private text group with other multilingual staff members in the region, where the group could support each other through the joys and challenges of providing multilingual care.

Participants also described situations where they forged close relationships with multilingual staff and families in a way that was beyond the expectations of their work. For example, Addy described how “With limited resources and a lot of… getting creative and going above and beyond our work tasks to really make things work for families,” her team collaborated and involved all members of the family, including siblings, to support the child receiving services. As a team, they developed a successful treatment plan that included video modeling, resulting in positive outcomes for the family, including injury reduction. Addy continued, “All of our ABA staff spoke Spanish, which was really helpful and so we could really have the caregivers, and everyone involved be able to communicate with every single person on the team, not just some, but everyone.” Addy described these actions as going above her typical work expectations to meet the needs of the family, and she believed their success would not have been possible without a collaborative and trusting relationship between the fully multilingual staff and the family.

Lastly, some participants described how their supervision practices were tailored to support multilingual staff. For example, Vicki discussed her supervision practices toward multilingual RBTs and those seeking certification as a BCBA, which developed over her decades of experience. She did not attribute her supervision practices to workplace expectations or norms, and she recognized the potential for multilingual staff to be tokenized and highlighted the importance of directly and openly addressing cultural and linguistic diversity in supervision:

For people who are working in a bilingual context, [it’s important] to be the one to start talking about [multilingualism]. Because it’s a thing that’s there. It’s an element that’s there, and to mention it, so that [supervisees] can feel comfortable to talk about it as well, if they want to, if they choose to. And to recognize how important it is for the family… And then, [it’s also important] not to pigeonhole [staff]. Because I know that I’ve worked with some staff where it’s so easy just to keep them speaking Spanish. And it’s great, but it’s not *all* they want…So, to just be careful of that, and to verbalize that, and talk through what they want to make sure they get what they need out of the position.

Vicki later described how she believes rejecting ‘colorblind’ ideologies is helpful when supporting multilingual staff, who are often themselves members of historically marginalized racial and ethnic groups. Similarly, Karee, who also has decades of experience in the field, shared how she actively embeds concepts of cultural competency into her supervision practices and directly talks about systemic challenges faced by families to her supervisees, saying:

Talking about the gaps in services for certain populations [is important], I do this early on in [staff] supervision because I can’t always change the minds or the behaviors of persons who are already certified, but I can influence the students [working toward their BCBA certification]. So that’s how I feel I’m challenging [inequities].

Karee and other participants shared similar beliefs about the importance of supporting new generations of ABA providers where linguistic and cultural diversity are built into their early days of training.

Overall, this theme demonstrates how participants navigated barriers to equitable ABA by innovating and creating new positive spaces. They also found it helpful to directly address cultural and linguistic diversity through their communication with families and colleagues, and when supervising students of behavior analysis.

## Discussion

The purpose of the present study was to gain a deeper understanding of the experiences and needs of ABA leaders related to supporting multilingual ABA staff. The findings are supported by existing literature, provide a novel expansion of the literature, and have important implications. In the focus groups and interviews, participants reported a range of experiences, challenges, needs that echoed those of multilingual professionals in ABA (Martin Loya & Meadan, 2024a, b), in mental health fields (Verdinelli & Biever, 2009) and in bilingual education (Amanti, 2019). First, findings largely support what we know about the experiences of ABA autism providers in the U.S., for example, stressing the need for more support and training to provide multilingual care (Beaulieu et al., 2019; Martin Loya & Meadan, 2024a, b). In addition, including monolingual behavior analysts with experience supporting multilingual staff adds a novel perspective that is important in research and practice. While specific demographic data remain unavailable for multilingual behavior analysts and similar professions, such as multilingual special educators, it is reasonable to assume multilingual behavior analysts are underrepresented in the field (BACB, n.d.), and evidence suggests that families with autistic children in the U.S. struggle accessing multilingual supports despite their desire for it (Durán et al., 2022; Papoudi et al., 2021). Professionals have reported challenges when there are cultural and language mismatches within their child’s clinical team, such as strained communication (Martin Loya and Meadan, 2024b) and potential for biases (e.g., early intervention providers reported biased attitudes towards heritage-language speaking caregivers that may have contributed to increased disparities in service access; Tomczuk et al., 2022). Therefore, exploring provider perspectives and experiences supporting multilingual staff is important to enhance our understanding of their needs in order to develop effective organizational supports (e.g., professional development).

In general, monolingual behavior analysts with experience supervising multilingual ABA staff shared many similar challenges experienced by multilingual behavior analysts (e.g., lack of resources or training in other languages), and they expressed the need for professional development opportunities related to multilingualism in ABA practice. The desire for more professional development is in alignment with the reported needs of special educators (Fowler et al., 2019) and Spanish-speaking BCBAs (Martin Loya & Meadan, 2024a). Participants also discussed how organization-level factors heavily influenced their experiences in providing multilingual care and supporting multilingual staff. These findings are supported by other research that demonstrates how leadership and organizational climate impact healthcare clinicians’ behaviors, well-being, and ability to provide effective care for multilingual children and families (Chavez et al., 2022; Gilin et al., 2023; Williams et al., 2022). Despite participants valuing multilingualism, some monolingual participants were also self-reflective and recognized their active participation in systems that could be discriminatory (e.g., recall Helen sharing how her place of work tokenized their one Spanish-speaking staff member, or Sophie’s comment about her workplace refusing services to heritage-language-speaking families). The disconnect between valuing multilingualism and having (or not having) the ability to provide high quality multilingual autism care is a tension that has also been reported by autism professionals in the United Kingdom ([U.K.]; Davis et al., 2024; Howard et al., 2024). Additionally, and of important note, most participants reported receiving no training to support multilingual staff in the context of their work, which is in alignment with findings in the U.S. (Martin Loya et al., 2025) and in the U.K. (Davis et al., 2024). Despite this, participants expressed a clear understanding of steps their leadership could take to support multilingual staff and provide the best care for multilingual autistic children and their families. However, they reported that organizations, especially large nation-wide businesses, often pose challenges in providing individualized support, such as English-only policies. BCBA participants in Martin Loya and Meadan (2024b) reported similar challenges working for companies with English-only policies that forbade them from providing heritage-language care for families. As mentioned, English-only policies may contribute to weaker bonds between caregiver and child (Oh & Fuligni, 2010), and heritage language maintenance is a protective factor for immigrant populations in the U.S. (Elshahat & Moffat, 2022; Garcini et al., 2021). Another important takeaway from the present study is the importance of using an intersectional theoretical lens from study conceptualization, recruiting research team members, collecting and analyzing data, and sharing findings. Both monolingual and multilingual participants identified biases and discrimination experienced by multilingual ABA staff, and we believe our purposeful recruitment efforts were successful when creating focus group and interview environments that encourage sharing of emotionally difficult topics such as discrimination and racism (experienced personally or witnessed by participants). Overall, participants reported many experiences that highlighted the need for more professional development opportunities, systemic and organizational-level change, and research on multilingualism, autism, and ABA using an intersectional theoretical framework.

### Implications and Limitations

This study presents important implications for practice, policy, and research, but it is essential to highlight that qualitative findings are not meant to be generalized. We recommend that readers use the following implications as a starting point to foster discussions and evaluate policies and practices within their own organizations. In practice, the critical role of organizational leadership cannot be understated. We recommend that organizations, both large and small, examine their policies to ensure they are not engaging in discrimination toward multilingual families (Kornack et al., 2019), nor multilingual staff. Of note, participants’ stories of multilingual staff frequently having their workdays interrupted to assist with unscheduled translation or interpretation tasks is concerning because frequent workday interruptions are linked to decreases in workplace satisfaction (Körner et al., 2024). To address such issues, leadership is encouraged to engage with organizational behavior management best practices to develop strategies for retaining and promoting talent from within (Akpapuna et al., 2020; Rosales et al., 2023). For example, we recommend that organizations leverage the diversity of their workforce by promoting from within, as direct care staff tend to reflect the population’s racial and ethnic makeup more accurately than those in leadership positions (i.e., behavior analysts; Rosales et al., 2023). Additionally, in alignment with experiences shared by the participants, we recommend organizations implement creative mentorship programs that provide tailored support for multilingual staff which may help with retention and encourage them to nurture and utilize their linguistic skills to support the population of multilingual autistic children in the U.S. that is projected to continue growing (Vespa et al., 2020).

As a field, we are encouraged to follow the example of many of the participants in this study and *Build Community and Create Solutions* to make change in policy and practice. We encourage active membership within relevant organizations to voice challenges and needs related to supporting multilingual staff. Participating as a subject matter expert, responding to questionnaires, and providing feedback are some ways that individuals can influence policies and practices within certifying bodies and professional organizations such as the BACB, Association for Behavior Analysis International, and Association of Professional Behavior Analysts. For example, subject matter experts for the BACB approved a change in continuing education policy to include Diversity, Equity, and Inclusion requirements for behavior analysts (BACB, 2023), to address an area of known need (Zarcone et al., 2019). Additional forms of advocacy and building community to promote policy changes are also important, for example, professionals in ABA have been found to seek support in anonymous online spaces (Price et al., 2024). Indeed, grassroots organizing can and does change the world; readers are encouraged to explore advocacy movements in disability support and education (e.g., Allen et al., 2024; Proctor et al., 2023) and engage in self-learning to build their knowledge and skills in advocacy to impact policy changes. Resources such as advocacy case studies and checklists (Edelson et al., 2021; Evanko et al., 2024; Thompson et al., 2023) can guide our learning. Advocacy is likely to play a key role in our ability to address additional structural barriers highlighted by participants, such as financial compensation for staff working across languages and establishing clear processes for collaboration between monolingual and multilingual staff. Structural barriers play an important role in our ability to provide effective care; for example, the autism screening and diagnostic process for Spanish speakers in the U.S. is incredibly challenging due in part to bilingual clinician shortages, which result in delays for Spanish-speaking families seeking support (Chavez et al., 2022; Morgan et al., 2024). To address these barriers, advocacy to enact policy changes may create a pathway toward more inclusive, equitable, and productive work environments, and we recommend providers remain engaged and connected with their local, regional, and nation-wide communities in autism care and support.

In research, this study underscores the need for further exploration and support in this area. In alignment with past recommendations, multilingual individuals are encouraged to lead research projects and work with interdisciplinary teams. Researchers are encouraged to tailor interventions and purposefully recruit multilingual professionals, autistic children, and their families, who have historically been underrepresented in disability research (Steinbrenner et al., 2022). Machalicek and colleagues (2022) provide recommendations that may assist in these efforts. When conducting professional development research, consider also engaging in open science practices so efforts can be more accessible to researchers and providers who may otherwise be trapped behind paywalls (Howard, 2019); indeed, multilingual behavior analysts have previously reported that more accessible research would be beneficial (Martin Loya & Meadan, 2024a). Researchers in a range of disability serving fields have offered rationale and guidelines to assist other researchers who want to engage with open science practices (Cook et al., 2022; Gilroy & Kaplan, 2019; Tincani et al., 2024).

This study had limitations. The first limitation was that one focus group had only two participants. Researchers recommend at least three people for a focus group to be successful (Tracy, 2019). While the two participants were heavily engaged in the conversation, it may have been more fruitful with at least one additional person. Another limitation of focus groups includes the possibility of participants coalescing around the same ideas due to social pressure (i.e., social desirability bias; Robinson, 2019; Tracy, 2019). However, in the case of the present study, all participants had similar education levels (graduate degrees), work experiences (supporting multilingual ABA staff), and were bound by the same Ethics Code (BACB, 2020; Robinson, 2019). Therefore, the opportunity for novel connections and interactions was believed to supersede the possibility that participants may coalesce around the same ideas due to social pressure or feel discouraged from sharing dissenting ideas. Lastly, the demographics of participants, while diverse in some areas (e.g., racial and ethnic diversity), were not diverse in other areas. For example, the entire participant sample identified as female, and we had an overrepresentation of Spanish speakers. While the BCBA population as a whole is majority female, perspectives from those who identify as male, non-binary, and speakers of other languages could have provided additional perspectives. Also, including additional interested parties in autism care, such as self-advocates or family members who are not BCBAs, may have illuminated additional novel findings.

## Conclusion

Little is known about the multilingual services autistic children and their families receive in the U.S., but early research demonstrates families value multilingual care (Durán et al., 2022). However, autism providers often struggle due to the lack of multilingual personnel and adequate training (Beaulieu et al., 2019; Martin Loya & Meadan, 2024a). The present study sought to build a deeper understanding of the experiences and needs of monolingual and multilingual leaders in ABA who support multilingual ABA staff. Findings from this study demonstrated how organizational policies can impact staff in positive ways (e.g., providing translation support, purposeful hiring, and culture-matching staff) and in ways that present barriers (e.g., the persistent shortage of multilingual personnel, and tokenization of existing multilingual staff). In addition, participants reported building community and creating solutions beyond the confines of their workplaces, such as starting their own businesses or private support groups, going above and beyond in their care and communication with staff and families, and providing tailored supervision to multilingual ABA staff. Providers and researchers are encouraged to examine their organizational policies and practices to promote equity toward multilingual autistic children, their families, and the direct care providers who support them.

## Electronic supplementary material

Below is the link to the electronic supplementary material.

Supplementary Material 1

## Footnotes

1 Previously referred to as English learners or bilingual (e.g., Martin Loya & Meadan, 2024a), *multilingual* is preferred strengths-based language and is more inclusive of various linguistic profiles (ASHA, 2023; Soto-Boykin et al., 2021).

2
*Latino/Hispanic* is an imperfect catch-all term for populations with diverse racial, ethnic, national, and gender identities. The authors recognize efforts toward more gender-inclusive language and believe these efforts are important (e.g., the use of Latinx or Latine). However, most Latinos/Hispanics do not use gender-neutral terminology (e.g., ABA participants in Martin Loya & Meadan, 2024a, b, and more broadly, Pew, 2024; Ramos et al., 2023); therefore, when using broad references, *Latino/Hispanic* will be used, and individual’s language preferences are maintained.

3 Notably, many participants used the words *culture* and *language* interchangeably. The word culture is maintained in the phrase *culture matching* to maintain the participants’ voices.
