# Supplementary material for: Experiences and Needs of Leaders Supporting Multilingual ABA Staff: A Qualitative Exploration
Source: J Autism Dev Disord. 2025 Apr 10;56(9):3585–99. doi: 10.1007/s10803-025-06816-w (PMC13427905; doi:10.1007/s10803-025-06816-w)
Supplement: Supplementary file 1 — Supplementary Material 1 [file 10803_2025_6816_MOESM1_ESM.docx]

**Experiences and Needs of ABA Leaders Supporting Multilingual ABA Staff: A Qualitative Exploration - Supplemental Information**

***Melanie R Martin Loya, Hedda Meadan, Elaine Macias Gilmartin***

**Table of Contents**

[Screening Form 2](#_heading=h.h2vlfunzhzhi)

[Demographic Questionnaire 3](#_heading=h.1fob9te)

[Focus Group Interview Protocol 8](#_heading=h.2et92p0)

[Focus Group Fidelity Checklist 10](#_heading=h.3dy6vkm)

[Code Book 11](#_heading=h.1t3h5sf)

[Credibility Measures and Quality Indicators 12](#_heading=h.4d34og8)

# **Screening Form**

Please answer these questions to determine your eligibility to participate in the BCBA focus group. If you are eligible, the form will continue to a consent form and conclude with a short demographic questionnaire.

Estimated time to complete all forms is **10 minutes.**

All questions with a red asterisk * are required.

Email*: _______________

**Screening Form**

First and last name:* _______________

**Are you currently certified as a Behavior Analyst (BCBA or BCBA-D) in good standing*?**
*

*To be considered in good standing, you should not have any disciplinary actions associated with your certification, as defined by the BACB.

-Yes

-No

**Please provide your certification number for verification. ***

Please ensure you enter the correct number.

_______________

**Have you been certified as a BCBA for more than one year?***

-Yes

-No

**Have you completed the 8-hour Supervisor training and are eligible to supervise RBTs or those seeking BCBA certification?***

-Yes

-No

**Are the primary recipients of your work children diagnosed with autism and their families?***

-Yes

-No

**Do you currently or have you ever provided supervision or mentorship to bilingual RBTs or bilingual BCBAs who practice bilingually or in their non-English language?***

-Yes

-No

**Are you over age 18, fluent in English, and do you live and work in the United States?***

-Yes

-No

**Are you willing to participate in a 1.5-hour focus group over Zoom with 3 to 5 additional BCBAs? ***

-Yes

-No

# **Demographic Questionnaire**

Thank you for agreeing to participate in this study! Please fill out this demographic questionnaire.

**Your age in years:***

**Which best describes you? Select all that apply.***

-Female

-Male

-Non-binary

-Transgender

-Prefer not to say

-Other: _________________

**Which best describes you? Select all that apply.***

-American Indian or Alaskan Native

-Asian

-Black or African American

-Hispanic or Latino

-Middle Eastern or North African

-Native Hawaiian or Other Pacific Islander

-White or European American

-Prefer not to say

-Other: ___________________________

**What generation are you in the U.S.?***

-First generation - I was born outside the continental U.S.

-Second generation - My parent(s) were born outside the U.S., and I was born in the continental U.S.

-Third generation - My grandparent(s) were born outside the U.S., and I and my parents were born in the continental U.S.

-Four or more generations in the U.S. or Unknown

- Other: _________________________

**If you are first, second, or third generation, what is your family's country of origin?***

If more than one, please list and separate with commas.

__________________________________

**If you are first generation, how many years have you been in the U.S.?***

If you are not first generation, please write N/A

________________________________

**Please indicate your level of certification:***

-BCBA - Masters level

-BCBA - Doctoral level

**Number years of experience in ABA, including paraprofessional, if applicable (Behavior technician or RBT level):***

**State where you practice ABA***

If you practice in multiple states, please choose the state where you predominantly practice.

-Drop-down selection with states

**Primary locations where your ABA services are provided*** Select all that apply

-Clients homes

-Schools - Early Childhood

-Schools - K - 12

-Clinic

-Other: ______________________________

**What is the primary age range of the children receiving services under your care?*** Select all that apply

- Birth to 3
- 3 to 5
- 5 to 7
- 7 to 9
- 9 to 11
- 11 to 13
- 13 to 15
- 15 +

**How would you best describe your current role?***

- I primarily work 1:1 with clients (i.e., I provide direct support to children, or their caregivers and I do not supervise RBTs or behavior technicians)

-I primarily provide **case management and supervision of RBTs or behavior technicians** (e.g., I might travel to individual client’s homes and/or schools to provide support or I stay within one clinical setting).

-Director - Local: I primarily provide **higher level clinical and administrative support within a single location** (e.g., one school or clinic, may also involve activities from Option 1)

-Director - Regional and beyond: I primarily provide **higher level clinical and administrative support across various distinct locations** (e.g., across various cities, may also involve activities from Option 1)

-Other: ______________________________

**Have you received any training or mentorship specific to supporting children on the autism spectrum from heritage language (i.e., non-English) speaking homes?***

-Yes

-No

**Have you received any training or mentorship specific to supporting bilingual staff who work with children on the autism spectrum from heritage language (i.e., non-English) speaking homes?***

-Yes

-No

**If you answered yes to one of the above questions, please select what training or mentorship you received specific to supporting bilingual children or staff.**

Select all that apply

-Direct supervision or mentorship from a bilingual individual related to providing bilingual care

-Attended a CEU event online or in person

-University coursework related to supporting bilingual children or staff

-Other: ______________________________

**Language(s) you have a working proficiency in:***

Select all that apply

-English

-Arabic

-Chinese - Mandarin

-Chinese - Cantonese

-French

-Korean

-Russian

-Spanish

-Tagalog

-Vietnamese

-Other: ______________________________

**What roles or identities do you hold related to the autism/autistic community? Select all that apply.***

-I identify as autistic/someone on the autism spectrum.

-I am a parent or caregiver of someone autistic/on the autism spectrum.

-I am a sibling of someone autistic/on the autism spectrum.

-I am the child of someone autistic/on the autism spectrum.

-I am a close family member of someone autistic/on the autism spectrum. (e.g., grandparent, aunt/uncle, cousin).

-I am a close friend to someone autistic/on the autism spectrum.

-I am in a romantic partnership with someone autistic/on the autism spectrum.

-I have no additional connections to autism outside of my profession.

-I prefer not to share.

-Other: ______________________________

**Captions will be automatically transcribed in the Zoom focus group. Do you require any additional accommodations to participate in the focus group?***

The researcher will email you directly to arrange.

-Yes - I need additional accommodations to participate.

-No - I do not need additional accommodations to participate.

# **Focus Group Interview Protocol**

**Introduction**

- Thanks for joining everyone! I am very excited to be here with you all today and grateful for sharing your time and expertise with me. To introduce myself quickly before we discuss the purpose and expectations for today: [introduction] I’m also a BCBA who practiced in the field for ten years before starting my doctoral program. This focus group is part of my dissertation research.
- As noted in the consent forms you have all signed, your participation today is voluntary, and you can end your participation whenever you’d like without penalty. This focus group will be recorded so that I can analyze the transcript when we finish. All data and information will be in a secure data storage location and de-identified to protect your privacy.

**Purpose**

- The purpose of this focus group is to learn more about your experiences, perceptions, and needs related to supporting bilingual staff, and by extension, bilingual or heritage-language-speaking families. I aim to develop training and resources to help bilingual ABA practice in the U.S thank you for being a part of this work!
- As a reminder, you will receive one 35-dollar gift card for your participation in today’s focus group and a second 15-dollar gift card after responding to a summary email you will receive by [DATE].

**Expectations and Norms**

- Mutual respect and confidentiality. In this space, we will continue to abide by our code of ethics, just as we do daily. Please do not discuss clients or employees using identifiable information. Also, please do not share identifying information about each other with friends or colleagues. The ABA world is small, so let’s be sure this is a safe and confidential space for everyone here. It’s important to me that participating in my research is a positive and meaningful experience for all of you. I need your support to make sure that happens.
- Please be mindful of sharing space with others. I ask that after each question, we all pause and think for at least five to ten seconds before beginning to talk. Additionally, if you have answered a question, please do not answer again until others can contribute. Before moving on to another question, I will ask if anyone else has additional comments. Everyone will be able to respond to all questions, and everyone’s feedback is essential and valued.
- There are no right or wrong answers. I want everyone to feel empowered to share their thoughts.
- Last, please state your first name (or pseudonym or nickname) prior to answering a question. This interview is being recorded and you stating your name will be very helpful when I transcribe the audio and analyze everything that’s been shared.

Are there any questions before we begin the focus group interview?

Let’s start with brief introductions. Let’s list our first name, role at work, and briefly note your experience either in working in other languages, or how you have supported employees or families who speak other languages. I’ll give a hypothetical to model what I am looking for: *“My name is Melanie. I am a clinical director at a small clinic in Texas. I work in English and don’t speak any other languages, but I have supported RBTs who provide bilingual ABA services.”*

**Knowledge**

1. What do you know about autism and bilingualism?

In the following questions, I will use the term *heritage-language* to refer to any language other than English, and I use the term *bilingual* to mean when both English and the heritage-language are used.

1. When providing bilingual or heritage-language services, what are some possible positive outcomes for:
   1. Children on the autism spectrum
   2. Families
   3. Bilingual staff who provide those services
2. When providing bilingual or heritage-language services, what are some possible challenging or difficult outcomes for:
   1. Children on the autism spectrum
   2. Families
   3. Bilingual staff who provide those services
3. What do you know about how to support bilingual staff so they can provide high quality services for non-English speaking families?

**Experiences**

1. What experiences have you had with training, mentoring, or other resources related to providing bilingual services?
2. What are some actions or policies you have implemented in your place of work to support bilingual staff who provide services for non-English speaking families?
3. Can you provide an example of a positive experience you’ve had working with or supporting:
   1. Bilingual staff
      1. Follow up – BCBAs or RBTs?
   2. Children and Families
4. Can you provide an example of a challenging experience you’ve had working with or supporting:
   1. Bilingual staff
      1. Follow up – with BCBAs? With RBTs?
   2. Children and Families

**Needs**

1. When you think about the experiences you have had in your workplace related to bilingual services, what would have been helpful for you to better support:
   1. Bilingual staff
   2. Children and Families

# **Focus Group Fidelity Checklist**

| FG Date: | Time Start: | | Time End: |
| --- | --- | --- | --- |
| Duration of FG in Mins: | | **# of Participants:** | |
| Participants Present (Initials): | | | |

| **BEFORE:** | **Complete?** |
| --- | --- |
| -Enable Captions |  |
| -Put Devices on Do Not Disturb |  |
| -Close doors, ensure privacy from household |  |
| **DURING**: |  |
| -Start recording |  |
| -Introduction of interviewer and participants |  |
| -Purpose of the project introduced |  |
| -Participants rights reviewed |  |
| -Review expectations of confidentiality in focus group |  |
| -Interview questions asked per interview protocol |  |
| -Participants asked about follow-up questions after interview concludes |  |
| -Participants are informed of member check process |  |
| **AFTER**: |  |
| -End Recording |  |
| -Save Captions |  |
| -Keep computer on and plugged in until saved confirmation |  |

| **Code Book** | | | |
| --- | --- | --- | --- |
| **Themes** | **Subthemes** | **Codes** | **Example Quotes** |
| **Organiza-tional Policies and Actions Impacting**  **Bilingual Staff** | Steps in the Right Direction | Training Received | We luckily have [multiple] BCBAs that are bilingual. And then we have current trainees that are bilingual as well…. Our clinical director is also bilingual. So, I feel like [they] helped me kind of even start advocating for that Hispanic outreach. |
|  |  | Agency Matters and Characteristics | One thing that organization I worked for adopted from my practices... They hired someone to facilitate community resources and they made sure to hire this person that had, I think she speaks 3 languages… And so that's something I saw implemented that helps a lot. |
|  |  | Advocacy | Letting them know that, you know, there's, extra effort that has to go into those families. And so, kind of making it an emphasis of like, this isn't just extra work, but it's making sure we're providing quality care to these families. |
|  | Barriers to Progress | Training (Not) Received | I also haven't had formal training, especially ABA specific, I guess, formal training… I did have to while I was working in the hospital system, we did have to do a little training about working with interpreters. … [but I received] nothing at all, ABA specific and really nothing at all specifically about bilingualism or promoting heritage language, or anything like that. |
|  |  | Agency Matters and Characteristics | Some centers don't take Medicaid, but there's parents still calling because they want answers, and they want resources. So, [admin] will go in the chat and they're like, is there anybody who speaks Spanish to take the call? So, it's never been, we need to hire Spanish speaking [staff]… It's just, you speak Spanish? It's expected for you to provide [Spanish] services. |
|  |  | Advocacy (with backlash) | We announced [an event for] clinicians of a [demographic] to support families of a [demographic]… and BCBA's are outraged that there is discussion about why these services are needed, or why materials have to be printed in heritage languages, and why these considerations are being made, and why there's even a discussion in ABA about these things. |
| **Building Community & Creating Solutions** | | Mentorship or Supervision of bilingual staff | When it comes to staff, I feel like you are able to build up a lot more of the rapport with the staff, but you get a lot more quality when you're meeting them in their heritage language, and giving them the instructions, and the procedures in a way that they could understand it a lot easier. And also respecting if they do want it in their heritage language. |
|  |  | Homegrown solutions and Community | [I have a text group of bilingual BCBAs who] all went to the same university, and I was their senior. So, I do help provide supports and they text me if they needed for something, especially if it's a translation thing. We work through it together if I can't figure it out. |

# **Credibility Measures and Quality Indicators**

| **Credibility Measures** | |
| --- | --- |
| Triangulation | Data were analyzed by a team of three experienced researchers. |
| Findings | Focus group transcripts were coded systematically and meaningfully by three experienced coders using reflexive thematic analysis (Braun & Clark, 2022). Coders met to discuss disagreements until consensus was met and a final codebook was created. Codebooks were used in a final round of coding using Dedoose (2022) by the first author. |
| Member Check | Following all interviews, member checks were conducted (Brantlinger et al., 2005). Data was summarized and sent to participants to accept, edit, or add to the summaries to verify their accuracy and authenticity. |
| Documentation | Throughout the process, ongoing detailed documentation of processes were captured to ensure sufficient trustworthiness and credibility. This occurred using a focus group interview fidelity checklist, notes taken during and after the focus groups and interviews, and ongoing detailed field notes documenting all procedures. |
| Reflection | The primary researcher and research assistant kept field notes using thick descriptions documenting the focus group and interview process. These notes included information that may not be apparent from audio or transcripts alone (e.g., participant emotions and researcher responses) that were important when analyzing P1 data and were combined with transcripts to provide deeper understanding. |
| Collaborative Work | Three experienced qualitative researchers worked together to analyze data to ensure results were not biased. |
| **Quality Indicators** | |
| Semi-Structured Focus Group + Interview Protocol | - Protocol questions and topics were reasonable to address the stated research question (RQ1, of the larger study). - Participants were represented respectfully and appropriately in the report and their confidentiality was respected. - Adequate and reliable methods were used to record and transcribe interviews. |
| Data Analysis | - Findings were analyzed in an organized and systematic manner using reflexive thematic analysis. - Researcher reflexivity was used to promote full transparency of personal and professional biases and worldviews. - Documentation thoroughly described procedures to promote credibility and trustworthiness. - Reporting included substantial quotes and interactions between focus group participants to substantiate conclusions. |
